# Informational challenges of dengue surveillance and response. A case study from India

**DOI:** 10.3389/fpubh.2026.1809584

**Published:** 2026-05-20

**Authors:** Vidya Sanap, Sundeep Sahay, Rosario Martinez-Vega, Satish Agnihotri

**Affiliations:** 1 Centre of Technology Alternatives of Rural Areas (CTARA), Indian Institute of Technology (IIT) Bombay, Mumbai, India; 2 Department of Informatics, University of Oslo, Oslo, Norway; 3 Department of Disease Control, Faculty of Infectious and Tropical Diseases, London School of Hygiene & Tropical Medicine, London, United Kingdom

**Keywords:** dengue, digital surveillance, health information system, India, outbreak response

## Abstract

**Background:**

While digital surveillance systems are being widely deployed for infectious diseases monitoring, yet their potential remains underutilized in low-and middle-income countries (LMICs). Dengue, a significant global public health concern is threatening over half of the world’s population and would benefit from the strengthening of supporting monitoring systems. India contributes roughly to one-third of global dengue cases, making it an important context to examine and mitigate informational barriers to digital surveillance. This paper investigates the informational challenges inherent in digital surveillance systems in India, where despite extensive digitization efforts, the results remain limited due to resources constraints, for example of the total sanctioned Integrated Disease Surveillance Programme (IDSP) district public health laboratories, only 13% are functional and four (out of 30+) districts lacking sentinel hospitals in the state which is our empirical site.

**Methods:**

Based on a qualitative research design, an empirical qualitative study was conducted between (2023 and 2025) in two high-burden districts in western India. Data were collected through 57 semi-structured interviews, direct observations, and extensive document review across multiple levels from the facility to district, engaged with dengue surveillance processes. Thematic analysis identified challenges in the current information workflow and the use of digital systems.

**Results:**

Key challenges identified were identified at levels of macro structures and micro processes, and their mutual influences. Challenges include: (i) fragmented information flows at multiple levels, with the separation of biomedical and social determinants of dengue, operation of dual and overlapping programmes of Integrated Disease Surveillance Programme (IHIP-IDSP) and the National Vector-Borne Disease Control Programme (NVBDCP); (ii) field staff facing community resistance, inconsistent laboratory protocols, reporting pressures, and reliance on informal communication that subverts rigid bureaucratic systems; (iii) emphasis on procedural targets over data quality, undermining the value of data for action taking.

**Conclusion:**

A clear gap was identified between the digital data infrastructures and field surveillance realities. Strengthening socio-technical interoperability, such as through harmonizing outbreak criteria across programmes and embedding socio-environmental determinants into routine surveillance could enhance dengue early warning and response.

## 1 Introduction

### 1.1 Dengue: the rising challenge

Dengue is one of the most significant mosquito-borne viral infection globally and a rapidly rising health concern. Over half of the world’s population lives in areas at risk of dengue transmission, and nearly 50% reside in endemic countries (1, 2). It is the only major communicable disease to have increased ten-fold from 2000 to 2019 (1), with Southeast Asia contributing approximately half of global cases (2). India bears a disproportionate share of this burden, estimated at 34% of all dengue cases (3), and continues to report annual increases, from 15,7,315 cases and 166 deaths in 2019 to 289,235 cases and 485 deaths in 2023 (4).

Historically considered a neglected tropical disease affecting marginalized populations, dengue has expanded globally due to increasing travel, trade, urbanisation, and climate change. This expansion has catalysed significant international attention and funding, including its designation under the International Health Regulations (IHR 2005) as a disease of priority concern (5), challenging its characterization as a “neglected” problem (6, 7).

In India, historically endemic states have seen a steady expansion of dengue transmission into peri-urban and rural settings (8). The economic burden of the disease is substantial, with global estimates ranging from US$8.9 billion to US$39.3 billion annually (approximately US$144 per symptomatic case). India shows one of the higher per case costs in South Asia, U$81.08, about 9% above the south-east Asian regional average (9).

Dengue is a mosquito-borne disease caused by four virus serotypes (DENV 1–4) of the genus *Flavivirus* and presents a wide clinical spectrum ranging from asymptomatic infection to severe dengue. Primary infection confers lifelong immunity against the infecting serotype but only a temporary immunity against other serotypes, making secondary infections more likely to result in severe disease due to antibody-dependent enhancement (5). *Aedes aegypti*, the primary vector, is active during daylight hours with a high affinity to human blood, and breeds in domestic water storage containers and other human-made sites. Structural and environmental factors such as unscreened housing, dense residential areas, poor waste management, sewerage, and lack of piped water supply, provide favourable conditions for sustained transmission (5, 10, 11). Climate conditions further amplify the risk: rising temperatures and increased rainfall extend mosquito breeding seasons, and expanding vector suitability into previously non-endemic regions (10).

Effective surveillance is central to dengue prevention and control, enabling early outbreak detection, trend monitoring, and targeted allocation of resources (5, 10). Rapid advances in digital technologies, such as geographic information systems (GIS), big data, cloud computing, smart phones, and artificial intelligence (AI) and machine learning (ML), are transforming disease surveillance, early warning, and response systems (12). Despite these innovations, implementation remains uneven resulting in fragmented information flows. World Health Organization’s (WHO) global digital surveillance system, DengueNet, has been used to gather data from member countries but is constrained by incomplete reporting and limited adoption (13). Many countries have developed national epidemiological databases, but often with limited integration of entomological, meteorological, socio-demographic, or environmental data sources (14–16). Notable exceptions, such as China, have successfully integrated heterogeneous surveillance data from multiple sources using ML-based analytics. However, these complex integrated systems are also susceptible to diverse socio-technical challenges, including bias in AI models, lack of transparency and explainability in model outputs, unequal use and access of digital across tools population, heterogeneous data formats, overfitting to historical patterns that misses emerging epidemiological trends, rapid shifts in environmental and sociodemographic factors, and poor data quality or timelines. Such constraints undermine the potential of digital technologies in strengthening early warning signals, digital contact tracing, and in the evaluation of interventions (17). Mobile-based surveillance platforms such as MOSapp, DISapp, and DengueChat have further expanded community-level reporting capacities. However, their impact remains constrained by persistent challenges, including data quality issues, model bias, limited interoperability, and low digital literacy (18, 19). More fundamentally, these tools tend to prioritize early warning signals and digital contact tracing, while largely overlooking the social and environmental determinants that shape both vector dynamics and host susceptibility. As a result, key socio-technical conditions influencing dengue transmission remain insufficiently captured and addressed within current digital surveillance systems.

Dengue transmission is affected by both biomedical and social determinants, raising the need for unified surveillance systems capable of integrating clinical, laboratory, entomological, environmental, and behavioral data. Such integrated systems can help better assess risk levels at granular geographic and population levels, enabling more equitable interventions (20). However, materializing these benefits in practice is a non-trivial challenge (21–23), arising from socio-technical conditions of diagnosis, reporting, vector control, and data integration. A major challenge is the systematic underreporting of cases, particularly due to the exclusion of private sector data due to various disincentives and constraints for reporting (5, 24, 25). Inadequate clinical reliability of data in the absence of laboratory confirmation limits accuracies in diagnosis (26–28). Entomological surveillance, remains labour intensive and costly, limiting its coverage despite the availability of emerging digital tools (20). Insecticide resistance among *Aede*s mosquitoes complicate vector control and requires robust monitoring frameworks (29). The WHO Integrated Vector Management (IVM) strategy promotes coordinated chemical, environmental, and biological interventions tailored to local contexts (30), yet implementation is weak due to poor intersectoral coordination between health, water, sanitation, and urban authorities (6, 7).

Given this background, the primary objective of this paper is to understand the drivers of weaknesses in dengue surveillance systems in selected sites in India, and to identify potential pathways for strengthening them towards integrated surveillance. This objective is met through two sets of analysis: (i) empirical assessment of the functioning of the dengue surveillance system on the ground, including macro level structural constraints and micro level surveillance practices; (ii) analysis of how the system can be strengthened through improved unification of the biomedical and social components of the surveillance system. The study employed a qualitative case study approach, using a macro–micro linkages framework to examine how interactions between institutional structures and field-level practices shape dengue surveillance and response system.

## 2 Materials and methods

### 2.1 Research context

In India, the administrative framework is delineated into interconnected vertical and horizontal entities: national, state, district, sub-district, rural villages and urban units, including municipal local bodies and wards. Although public health is primarily a state responsibility, dengue surveillance is governed nationally through two programs: The National Vector Borne Disease Control Programme (NVBDCP) and the Integrated Disease Surveillance Programme (IDSP). While both are under the common umbrella of the Directorate General of Health Services, Ministry of Family Health and Welfare, they function relatively independently with parallel information flows (31, 32).

The 2015 mid-term review of the IDSP programme highlighted the need for strengthening epidemiological data collection, reprioritization of diseases, and improvements in ICT infrastructure and human resources. The review highlighted the necessity for capacity building, integration, intersectoral collaboration, standardization of laboratory tests, and increased political support (32). This review catalysed the government to revamp the entire functioning and structure of the IDSP (31), leading to the transition in 2018 from aggregate paper-based system to real time case-based system on the Integrated Health Information Platform (IHIP), now formally known as IHIP-IDSP.

### 2.2 Study design

This study employed a qualitative case study design to enable an in-depth examination of how dengue surveillance functions across governmental units, levels, and programmes. Fieldwork was conducted between November 2023 and March 2025 in two high-burden districts of a western Indian state. The contours of the research design are elaborated below.

#### 2.2.1 Site selection

We selected a high burden state (anonymized to protect confidentiality), based on an analysis of data from fifteen high burden states, which together accounted for over 80% of the country’s mortality and morbidity (33). The selected state is one of India’s most urbanized states in the country, with a high overall disease burden and pronounced intraregional disparities (34). The major vector-borne diseases prevalent in the state include dengue, malaria, and chikungunya, reporting a high proportion of dengue cases and deaths nationwide (4).

The research was conducted by four authors, with the lead being formally engaged with the State Health Department in the capacity of an independent university researcher, and the other three authors bringing research expertise in Malaria and Health Information Systems, without directly participating in primary data collection. As a project staff, the first author had previously engaged with the state authorities in strengthening their digital interventions, outside the domain of disease surveillance. This prior engagement and university affiliation enabled her obtaining formal permission and research access from the state. This permission allowed her to access IHIP-IDSP data for dengue for the last 2 years, which provided the empirical basis for comparative analysis across the selected two high burden districts. In consultation with the state, in each of these two districts, three rural primary health care centres (PHC) were selected, including two where a recent dengue outbreak had occurred.

#### 2.2.2 Participants and sampling

Purposive sampling was used to identify stakeholders involved in dengue surveillance from the community to the district levels. The first author conducted initial exploratory field visits to identify and map key stakeholders involved in dengue surveillance and response, enabling a multi-level analysis of surveillance practices.

The district-level administration is responsible for conducting surveillance activities through their rural PHCs and sub-centres (SC), coordinated by the District Health Officer (DHO). Secondary healthcare facilities at district and sub-district levels (called the Block) are headed by the Civil Surgeon, and tertiary care health facilities (affiliated to medical colleges) are under the Directorate of Medical Education and Research. For this study, a total of 57 participants within the district level were included, comprising community health workers, Clinical staff, administrative staff and technical support staff. The following table details the staff details under each category:

Community Health Workers: Accredited Social Health Activist (ASHA), Multipurpose Health Workers Male [MPHW (M), Auxiliary Nurse Midwifery (ANM)].

Clinical Staff: Medical Officer (MO), Pharmacy Officer, Lab technician, Health Assistant (vector borne disease).

Administrative Staff: District Surveillance Officer (DSO), District Malaria Officer (DMO), Block Health Officer, Block Health Assistant (vector borne disease), Insect Collector, IDSP Data Manager; Lab Scientific Officer (LSO).

### 2.3 Data collection

This took place across a wide spectrum of relevant staff involving different methods, as summarized in the Table 1 below.

**Table 1:** Summary of data collection.

Level	Stakeholders	No of interviewees	No of interviews	Observations (in hours)	Key issues for discussion
Public Health Facility	Multipurpose Health Worker (male)/MPHW (M)	8	12	50 h	1. Functioning of active and passive surveillance 2. Outbreak detection and response process 3. Data reporting practices and use
	Health Assistant (vector borne diseases) (MPHW supervisor)	6	7		
	Pharmacy Officer	5	5		
	Lab Technician	6	6		
	ANM	2	2		
	ASHA	5	5		
	Medical Officer	6	3		
Block	Block Health Officer	2	2	4 h	1. Supervision of surveillance and response 2. Data analysis and use
	Block Health Assistant (vector borne diseases)	3	3		
District	District Surveillance Officer	2	2	30 h	1. Monitoring and Implementation of surveillance programmes 2. Data reporting and analysis, its use 3. Co-ordination between NVBDCP and IHIP-IDSP 4. Confirmatory lab testing
	District Data Manager	2	3		
	District Malaria Officer	2	2		
	Lab technician (District Sentinel Hospital)	3	3		
	Insect Collector	3	3		
	Lab Scientific Officer	2	2		

A total 60 formal interviews were conducted for 57 participants, including some repeat interviews. Additionally, there were various informal meetings and discussions, particularly at the time of in-site observation activities.

As summarized in the Table 1, data collection involved multiple modes including semi-structured interviews, non-participant observation, shadowing, and document analysis. These covered issues of reporting practices around outbreak detection and response, and factors influencing dengue transmission and the use of digital systems Non-participant observations were undertaken at health facilities and sentinel hospitals focused on understanding how field staff conducted routine practices related to data collection, reporting, laboratory testing, digitization, and data analysis. Shadowing of MPHWs (M) provided insight into routine practices surrounding community-level surveillance protocols, operational constraints, interactions between health workers and community members, and the conduct of container surveys. A total of about 84 h were spent on observation and shadowing. Additionally, secondary data was collected in the form of NVBDCP and IDSP guidelines and facility level dengue reports, which helped develop a more nuanced understanding of how nationally mandated guidelines were being implemented or not in practice. As we reached the stage of no novel insights were forthcoming through continued interviews, we reached a stage of “saturation” and moved to the stage of data analysis.

### 2.4 Data analysis

A thematic analysis process, as defined by Braun and Clarke (35), was developed to identify key themes emerging from the data. This involved understanding both structural conditions and micro-level practices and interpretively understanding how they mutually influenced each other. The following process was undertaken. First the data was grouped based on the three core phases of dengue surveillance: data collection, data reporting, and data analysis and use. Within each phase, codes were developed to capture existing practices, deviations, operational barriers, and informal adaptations. This was followed by a cross-theme analysis to identify similarities and differences in meanings to develop broader conceptual categories, at two analytical levels of micro-level practices and macro-structures shaping these practices such as programme governance and existence of material and geographical constraints.

To enhance analytical clarity, we explicitly distinguished between data derived from direct observations, interviewee accounts, and document review. While the study of guidelines across the programmes allowed us build insights to structural factors, observed practices and reported experiences of the respondents through interviews and observations helped interpret how the structural conditions influenced the shaping of micro-level practices of the staff in the field. The authors independently read the interview transcripts and developed their interpretations, which were then jointly discussed to develop integrated inferences.

As an example, we illustrate the analysis process, spanning syndromic, presumptive, and laboratory surveillance. We identified key challenges related to structures of inadequate testing infrastructure and resources, geographical constraints including transportation, and how these shaped practices relating to weak adherence to guidelines, subjectivity in interpretation of test reports, and use of different criteria for conducting tests. These issues were termed as first-level, codes which were grouped to define their interlinkages shaping “fallacies in the pathological confirmation of the diagnosis”. Similarly, at the facility level, we identified themes such as limited coordination between pharmacy and lab technicians, poor enforcement and supervision, and the separation of reporting from private sector facilities from the state systems. At the community level, we found that Excel-based reporting of container and adult surveys data, the absence of a software contributed to siloed reporting of “S,” “P and L” forms. Similar first-level codes were thematically grouped to define the second-level code as “lack of integration of biomedical and social determinant of dengue surveillance data. These micro level practices were interpreted in the context of macro structures of governance systems of the two national programmes, infrastructural deficits, community resistance and more.

## 3 Results

To address the research questions, findings are presented in two sections. First, a situation analysis describing how the surveillance is organised and functions across levels. Second, an analysis of cross-cutting informational challenges of fragmentation and approaches towards their unification.

### 3.1 Situation analysis

#### 3.1.1 Administrative structure and responsibilities

Based on stakeholder mapping, document review and empirical investigations, the formal administrative structure of the dengue surveillance programmes across community levels to districts is summarized in Table 2.

**Table 2:** Administrative structure for dengue surveillance.

Level	Head and supporting staff available at the specified level	
District	*DHO*	
	DMO—NVBDCP	DSO—IHIP-IDSP
	Insect Inspector	Data Manager
	Lab scientific officer	Epidemiologist
	Data entry operator	
	Supervisor	
Block	*THO*	
	Taluka Health Assistant (vector-borne diseases)	
	Taluka Health Assistant (waterborne diseases)	
	Data entry operator	
PHC	*MO*	
	ANM	
	Lab technician	
	Pharmacy Officer	
	Health Assistant (vector Borne diseases)	
	Health Assistant (waterborne diseases)	
SC	*Community Health Officer (CHO)*	
	MPHW (M)	
	ANM/MPHW (F)	
	ASHA	

A study of the administrative structure revealed that different resources were designated for monitoring and implementation of NVBDCP and IHIP-IDSP programmes in the district units. At the sub-district and facility level, the same health personnel were seen to be responsible for executing surveillance activities and conducting data entry for both surveillance programs. At the SC level, both male and female multipurpose health workers were available. The MPHW (F), commonly referred to as ANM, was responsible for maternal and child health services as well as family planning related programmes and activities aimed at prevention and control of communicable diseases.

As observed on the field, NVBDCP deployed dedicated personnel at the facility level, recognized as “state level” staff, such as lab technicians, health assistants (vector-borne disease), and MPHW (M) for epidemiological and entomological surveillance. In facilities where such deployment was absent, personnel were typically assigned by the district council or urban local bodies. We observed that the district-level NVBDCP programme office was commonly referred to as the “Malaria office” and headed by the “DMO.” The NVBDCP programme initially focused on Malaria and was subsequently expanded to include other vector-borne diseases and renamed as the NVBDCP in 2003. Despite this change, it was observed that old naming conventions were still followed at the district level, with a continued special focus on Malaria.

#### 3.1.2 Dengue surveillance and response system

The information workflows and resources under the two programmes is summarized in Table 3.

**Table 3:** Structure of dengue surveillance under IHIP-IDSP and NVBDCP.

Level	IHIP-IDSP		NVBDCP	
	Task	Responsible Actor	Task	Responsible Actor
Community	Syndromic surveillance (S form—digital reporting daily basis)	MPHW(M)	Larval surveys (paper-based weekly reporting—entomological surveillance)	MPHW(M)
Public Health Facility	Presumptive surveillance (P form—digital reporting daily basis)	Pharmacy Officer	Disease surveillance (paper-based weekly reporting—epidemiological surveillance)	Health Assistant (vector-borne disease)
	Laboratory surveillance (L-form daily digital reporting if rapid test kits are available)	Lab technician		
District	Monitoring and supervision	DSO and District Data Manager	Adult mosquito and larval surveys	Insect Collector
			Monitoring and supervision	DMO
Sentinel Hospital	Laboratory Surveillance (L-form daily digital reporting) ELISA IgM/NS1 test	Lab technician	Laboratory Surveillance (paper-based reporting—ELISA IgM/NS1 test)	Lab technician and Data entry Operator (if available)
Outbreak criteria	Based on the identified baseline values and threshold levels for each disease in the district (Threshold levels for each condition/disease to be set by analysing the previous 3–5 years of data by the district)		If 5 or more cases are positive during a period of 7 days in a village	
Outbreak Reporting	Event Alert (digital reporting) or Health conditional alerts (automatically generated)	Event alerts reported by the community, or any Health staff except the lab	Outbreak preliminary report (paper-based)	Health Assistant (Vector-borne disease)
	Outbreak report (digital reporting)	Disease Surveillance Officer	Outbreak final report (paper-based)	Health Assistant (vector-borne disease)

##### 3.1.2.1 The reporting process

This section outlines the dengue data reporting workflow, synthesized from field observations, interviews with frontline health personnel, and official programmatic guidelines. Dengue surveillance comprised of three components: syndromic (S), presumptive (P), and laboratory-based (L) surveillance. Syndromic surveillance was conducted by the MPHW (M) to actively identify suspected cases in villages based on symptoms such as fever, chills, cold, and cough. The MPHW (M) followed a 10-day monthly work schedule to complete household surveys in their assigned villages. ASHA and ANM also notified the MPHW (M) of suspected fever cases identified during their routine village outreach activities. However, online reporting through the IHIP Mobile application was done exclusively by MPHW (M). All fever cases were routinely screened for malaria by an MPHW (M), ANM, or ASHA and sent to the PHC for microscopy test. Negative malaria cases were not further diagnosed for any other arbovirus diseases. Whole blood samples for dengue and other arboviral testing were collected through hand venipuncture only when clusters of two to five fever cases were identified within the same village or neighbourhood, based on field-level judgment.

Prescriptive surveillance was conducted at the facility level by medical officers. During the monsoon season, blood samples were collected from nearly all fever cases and sent for confirmatory testing for dengue and other arboviral infections. As informed by staff, during inter-epidemic periods, samples were sent selectively, based on clinical assessment of symptoms and fever duration. Laboratory surveillance was also facility-based relied on the availability of rapid diagnostic test (RDT) kits and the transportation availability of serum samples to sentinel hospitals for confirmatory diagnosis. Both field observations and interviewee accounts indicated that, due to staff shortages, blood samples were most often physically transported by MPHW (M). RDT results were not considered reliable by the NVBDCP; therefore, even RDT-negative cases were often referred for confirmatory testing based on clinical presentation. At the district level, ELISA IgM or NS1 testing was performed according to the interval between fever onset and sample collection. Positive ELISA results were communicated promptly via WhatsApp to the relevant PHCs which then initiated contact tracing, travel history assessment, and additional sample collection. For official reporting, NVBDCP included only dengue cases confirmed by ELISA testing (31). Document review indicated that, although 51 sentinel hospitals were expected to be functional, four of the 36 districts lacked sentinel coverage (36). Although district public health laboratories (DPHLs) were established under IHIP-IDSP, they were functional in only three districts (37).

The findings suggests that the lack of community-level dengue diagnostic kits and standardized field-level clustering criteria renders dengue surveillance largely passive, potentially leading to underreporting. In addition, reliance on sentinel hospitals delayed processes of confirmatory diagnosis, particularly for remote rural facilities and in those districts where sentinel hospital coverage was lacking.

##### 3.1.2.2 Outbreak detection and response process

This section details the dengue outbreak detection and response process based on the analysis of the five outbreaks observed and field level consultation. ELISA-confirmed results were used for outbreak detection in both the NVBDCP and IHIP-IDSP programmes, although under different protocols. In NVBDCP, an event alert was generated when two dengue cases occurred within a contiguous cluster or when a single case of dengue haemorrhagic fever or shock syndrome was identified. An outbreak was officially declared when five or more individuals test positive within a village or when a dengue-related fatality occurred (38). In contrast, IHIP-IDSP defined outbreaks based on thresholds derived from the preceding 3–5 years of district-level data (39). In practice, however, the NVBDCP criterion (five or more cases or a single fatality) was applied by both programmes as observed during the analysis of five outbreaks, with preliminary outbreak declaration and closure reports were prepared in paper format and shared with district malaria and the IHIP-IDSP office via email, WhatsApp, and courier services. Although an event alert generation functionality existed in the IHIP-IDSP portal, its use was inconsistent, and alerts generated only after formal outbreak confirmation by the District Surveillance Officer. As observed in one PHC, this process did not always function as intended, with staff unaware of who had reported the outbreak despite being reported in the portal.

Following an outbreak declaration, response activities included container surveys, source reduction, fogging and spraying, and community awareness campaigns. It was observed that additional staff from neighbouring facilities were often mobilized to support epidemiological and entomological surveillance. PHC laboratory technicians conducted rapid NS1 antigen testing in the field, while complete blood count tests were performed by state-contracted private laboratories. ELISA testing was typically discontinued after an outbreak declaration. PHCs also coordinated with local political units (Gram Panchayats) for vector control activities, and district-level staff provided supervision and technical support. An outbreak was considered closed when no new cases were detected for 15 consecutive days. Under NVBDCP, a physical outbreak closure report was submitted to the district malaria office, while in IHIP-IDSP the data manager submitted a closure report authorized by the District Surveillance Officer. The physical reports included detailed information on dengue cases, surveys conducted, samples sent to the National Institute of Virology, control measures, and outbreak causes. This represented much greater level of detail than that captured in the IHIP-IDSP digital reports.

### 3.2 Thematic analysis of challenges in field-level surveillance practices

The analysis is presented under four thematic challenges identified through the analysis.

#### 3.2.1 Limitations of community level surveillance

##### 3.2.1.1 Syndromic surveillance narrowly captures febrile illness

Dengue typically presents as high fever accompanied by at least two symptoms including rash, severe headache, retro-orbital pain, myalgia, or arthralgia (40). However, it was observed that IHIP-IDSP syndromic surveillance captured only three categories (fever, fever more than 7 days and fever with rash). On the field, fever cases detected by MPHWs (M) were screened for malaria via a peripheral blood smear, and submitted to the PHC for microscopy testing, with only malaria-positive results communicated for follow-up. Malaria negative cases were not further investigated for other arbovirus infections. Laboratory screening for other diseases, apart from malaria, occurred only when clusters of fever cases were identified. MPHW (M) sometimes provided basic symptomatic treatment (e.g., short paracetamol courses) to fever patients with advice to attend a health facility in case symptoms persisted. Thus, many probable dengue cases never entered the surveillance system due to the overlapping symptoms, unavailability of community-level testing kits, and clustering criteria for fever cases, which distorted the baseline threshold and delayed cluster recognition.

##### 3.2.1.2 Community resistance and structural barriers to larval surveys

Larval indices were determined through larval surveys conducted by MPHWs (M) by inspecting the water containers of 10 households daily for signs of mosquito breeding. These surveys enabled the calculation of larval indices—House index^1^ (HI), Container index^2^(CI), and Brutu index^3^ (BI). According to the NVBDCP operational guidelines, an area was classified high risk for dengue if the HI or CI exceeded 10%, and the BI 50%. However, staff reported resistance from the community to have household entry to conduct the survey. A worker told us:

*During routine inspections, the community generally does not permit us to enter their homes to check for breeding sites such as refrigerator trays, flowerpots, and water vases. However, in the event of an outbreak, the community is more likely to grant access, either due to the impact of the infection or interventions by the Gram panchayat (the political body). There is also resistance to indoor fogging, which complicates efforts to control adult mosquitoes indoors.*

While doing container surveys, if the MPHWs (M) found larvae, they asked household members to empty the containers and dry it under the sun before use. However, this request was commonly not accepted due to prevalent water scarcity issues. A MPHW (M) said:

*Due to water scarcity, individuals are often unwilling to empty containers. They frequently promise to do so at a later time, which rarely happens. During outbreaks, we take the initiative to empty containers despite community resistance. We use temephos, a larvicide, to treat the water, rendering it unsuitable for drinking but acceptable for other domestic uses. Typically, on a routine basis, we avoid using this, as you never know if they use it for drinking, we get in trouble. Therefore, we reserve its use for public places only*.

The conduct of surveys was affected by insufficient and overburdened human resources. While government norms specified that one SC should cover 3,000–5,000 population in tribal and non-tribal areas, in practice, they catered to 10,000–150,000 which greatly burdened the conduct of surveys. This weakness of active surveillance was also acknowledged by NVBDCP whose IHIP website said: *Active surveillance is carried out by health workers through a fortnightly unit. However, due to the strength of health workers active surveillance is not carried out as per norms of Govt* (41). This limitation was also reflected in the container survey reporting. We checked three container survey reports, and found that for the last 3–4 months, no larval indices (HI, CI, BI) exceeded the threshold values. This issue was also raised by the district officer during their surprise supervisory visit to one PHC. The block health officer, the Disease Surveillance Officer, and the District Malaria Officer expressed surprise and believed it led to underreporting by MPHW (M) to avoid extra work if they reported high values for larval indices.

##### 3.2.1.3 Lack of attention provided to adult mosquito surveys

According to the NVBDCP mid-term plan for Prevention and Control of Dengue & Chikungunya, “adult collection is not envisaged in endemic areas as the adult may be infected with the virus” (42). The NVBDCP Compendium on Entomological Surveillance and Vector Control in India reported: “as there is no prophylaxis for dengue or other viruses transmitted by Aedes mosquitoes, it is highly desirable, for ethical reasons, that adult captures of Aedes vectors should be based on ‘landing collections’ only with instructions to avoid being bitten by mosquitoes”. In the landing collection method, mosquitos are collected using an aspirator when they attempt to bite exposed skin, with results reported as landing counts per man-hour (43). However, field observations indicated that Insect Collectors primarily did mosquito collections in resting positions (expressed as the number of mosquitoes collected per 2.5 h) using a torch and an aspirator in indoor locations including in closets and other sheltered sites.

Insect Collectors informed us they selected villages based on high values of the Annual Parasite Index (API) based on previous years outbreak data, an accepted measure of malaria transmission, which was given first preference. However, if the API was seen to be low, they selected villages where other priority diseases outbreak had occurred. According to an Insect Collector, adult mosquito collection data were used mainly for site selection and research testing rather than for routine surveillance. They also indicated their inability to conduct early-morning resting collections due to limited transportation availability and only selected villages having good road connectivity.

#### 3.2.2 Differential reporting practices and laboratory variations

##### 3.2.2.1 Fragmented data flows

Dengue surveillance data were reported differently by the NVBDCP and IDSP programs. Under NVBDCP, epidemiological and entomological data were aggregated weekly on paper and submitted to the district office. In contrast, the IHIP-IDSP collected case-based real-time data under S, P and L surveillance forms. Within IHIP-IDSP, data from P and L forms were automatically linked only when diagnostic tests were recorded in the clinical diagnosis form. In practice, however, limited coordination between pharmacy and laboratory staff meant that forms were often completed independently, resulting in duplicate entries and additional workload.

For confirmatory diagnosis, samples were sent to a sentinel hospital, where separately a laboratory diagnosis (L) form was completed separately not linked to the corresponding S or P forms. At the facility level, febrile routinely underwent complete blood count (CBC) testing conducted by a private laboratory contracted by the state government. These results were entered into the laboratory’s own digital portal and not integrated with either the NVBDCP or IHIP-IDSP systems. This fragmentation of data systems limited the ability to determine whether dengue infections were primary or secondary, an important distinction to identify risks emerging from secondary infections. In addition, under NVBDCP, entomological surveillance data were recorded exclusively on paper or in paper registers and excel files, independent of the digital systems of both programmes.

##### 3.2.2.2 Variation in laboratory testing and interpretation

Field observations and interviewee accounts revealed variations across laboratories, reflecting inconsistent application of national guidelines, which stated: “while sending the samples for lab confirmation, the day of onset of fever and day of sample collection should be mentioned to guide the laboratory for the type of test to be performed [NS1 for samples collected from day 1 to 5 and IgM after 5 days” (40)]. In practice, however, different testing criteria were seen to be followed. At the district civil hospital, ELISA NS1 testing was performed for all the hospital’s suspected outpatient department (OPD) patients under the assumption that the patient had visited the clinic in the early phase of the illness. IgM ELISA was performed for all blood samples received from other health facilities. A laboratory technician explained: “*MPHW (M) informs us if they have taken a sample in the early phase of illness, then we perform ELISA NS1*.” It was also observed that referring facilities did not record the date of fever onset while sending samples to sentinel hospital. At the municipal hospital, rapid NS1 testing was conducted for all OPD samples, and ELISA IgM or NS1 testing subsequently performed for positive cases depending on the availability of test kits rather than on clinical timelines. While noting the issue of missing fever onset data in records, one microbiologist justified by saying that: *The patient gets symptoms after 3–4 days of the mosquito bite, then they come to the clinic after 1–2 days. This already makes the time period more than 7 days till the blood sample reaches us*. This suggests ambiguities and misunderstanding of the criteria guiding laboratory test selection.

Overall, the lack of systematic information on fever onset, combined with non-standardized testing protocols and logistical constraints, limited effective implementation of national diagnostic guidelines.

Subjective differences in the interpretation of ELISA results further contributed to variability in reporting. For IgM ELISA tests, laboratory technicians interpreted results based on optical density values generated by the ELISA reader, using locally defined “grey zones” to classify results as positive, negative, or equivocal. One technician reported: *We declare the result positive if the value is on the borderline of the positive range. The intention is that the patient should get treatment and not to increase cases of any specific PHC*. Another technician said, *We reject test values if they are outside the defined range and ask to submit the samples after seven days again*. In contrast, another microbiologist stated: *I always show the results to our head. S/He checks the colour of the sample and declares the result positive if the optical density is within the +0.5/−0.5 range. In a positive test result, the sample has a yellow colour*.

These differing interpretative practices introduced further inconsistencies in laboratory reporting, magnified by laboratory constraints and variability in testing quality. Lab technicians also reported challenges faced by them includes inadequate maintenance support of ELISA equipment, including unreliable battery backup, inconsistent reagent supply, lack of functional air conditioning, insufficient consumables, and limited access to distilled water. Health staff sought to mitigate these constraints locally by reallocating maintenance grants or creating technical workarounds, such as programming ELISA protocols into blood bank equipment to avoid delays. During field visits, ELISA washers were non-functional in two of the three sentinel hospitals. As a result, washing steps—critical for removing unbound antigens or antibodies between incubation stages—were performed manually by laboratory staff.

At the facility level, additional variability was observed in the use of rapid NS1 diagnostic tests. Although manufacturers’ specifications allowed the use of either whole blood or serum, some laboratory technicians preferred serum due to concerns about false-positive results when using whole blood. One technician explained: *In serum, there is no RBC interference. In the RDT kit, we must use the same proportion of serum/whole blood and buffer. But we have to use an extra buffer for whole blood. Otherwise, it clots and gives incorrect results. The test result display area becomes invisible due to the red colour. In such cases, the weakly positive line is not visible, or sometimes, due to clotting, we may get a false positive line*.

#### 3.2.3 Limited value perceived of formally designated digital tools

Across sites, informal communication tools—particularly WhatsApp—were used more extensively than government-mandated digital portals. WhatsApp functioned as the de facto coordination layer for reporting OPD data, monitoring activities, emergency communication, sharing government orders and meeting circulars, and rapidly communicating positive confirmatory test results. Most health officials were members of five to six WhatsApp groups, including of private hospitals, effectively bypassing official reporting channels. One medical officer showed the researcher a WhatsApp group of private lab technician and private doctors through which they shared information about dengue patients. While this practice improved speed of communication, it contributed to data fragmentation.

Digital tools were used to enhance reporting compliance rather than promote local action. While discussing with the DSO about monitoring, s/he opened the IHIP-IDSP portal and said:

*This is our daily evening activity, let us see yesterday’s report. This is how the report look. It showed how many health facilities have completed S, L, P form reporting. Look the overall score is 94%. Our data manager daily by 5 p.m. download this report and send it over WhatsApp group and ask them to fill the data. We have separate group of MPW, lab technicians and pharmacy officers, THO and HA. Further THO and MO do follow up ahead.*

Although the IHIP-IDSP Training of Trainers manual assigns responsibilities to facility- and district-level staff for data quality checks, trend analysis, disease-specific threshold analysis, and public–private data comparisons (39), none of the districts conducted such analysis. Instead, we saw emphasis being placed on achieving reporting targets for S, L and P forms, respectively fixed at 92, 90, and 80%. The IHIP-IDSP manager said s/he regularly shared reporting status over WhatsApp by 3/5 pm to prompt report submission. Despite this, reports were often delayed or incomplete, and discrepancies between paper-based and digital reports were common.

A district surveillance officer described how these constraints affected reporting quality: “*We have a shortage of human resources. We also have facilities where OPD goes above 100 or 200. Also, in tribal areas, internet connectivity is an issue. Still, we encourage our staff to make the data entry at least for symptoms/health conditions listed in the IHIP-IDSP portal.*”

After verification of the “facility monitoring roster report” on the IHIP-IDSP portal, it was found that the quality score used for facility monitoring based on S, L, and P form reporting did not include a check for quality.

- Consistency Score—% Reporting Units who reported during the month, calculated on a daily basis, inclusive of nil reporting (adapted to a maximum score of 70 points).
- Quality Score—% Reporting Units who reported at least one case during the month, calculated on a daily basis (nil reporting excluded)—maximum score 30 points (44)

Even the state funding mechanism emphasized performance on reporting completeness including of outbreaks. State targets for the financial year 2025–26 were 99, 97, 95, and 92%, respectively (45). These forced the review meetings to focus on progress towards achieving targets over use of data for local action to mitigate the dengue burden. While discussing state level monitoring, a DSO said:

*State monitoring meeting largely focus on the reporting targets as well as the outbreak responses. In software, outbreak response is calculated at different stages, how quickly RRT is composed and action updated by RRT, when line list is updated by health facility, all this need to be updated on time in the portal. If we delayed in generating event alert or reporting cases in outbreak then outbreak response score is affected. Most of the time physical activity on ground is already started, even physical reports are updated. But we missed to update it in the portal which affects our outbreak response score. Now thing which are in my hand I tried to do on time such as outbreak closure. Based on this score, state tracks the top performing districts and bottom performing districts. This creates competition among district to be in the top 10. So, all DSO tries to improve their performance. DHO has also ranking every month. This programme wise points are also included in it. If score is low DHO ranking also get lower. That’s how it gets importance from everyone*.

We studied five outbreaks and cross-referenced with IDSP weekly output reports (46) which contained state wide outbreak details along with a column describing comments/action taken. We identified in these reports a primary focus on routine responses in terms of activities of fogging, active surveillance, source reduction, campaign-based programmes of community awareness. We assessed these reports from 2012 to 2024 and found a similar pattern of focus on routine activities, which was also confirmed through our empirical investigations.

While health workers found value in the use of digital tools to make their work visible to authorities, they found it limited in providing support to strengthen local action. The structural pressures of meeting compliance targets seemed to override focus on strengthening treatment protocols and treatment outcomes.

#### 3.2.4 Difference between physical realities and digital reporting

We observed multiple factors contributed to discrepancies between physical realities and their representation through digital reporting, including the detection of asymptomatic cases, prioritization of data reporting over care processes, engagement with human and material constraints. Institutional pressures were often seen to shape underreporting of data and continued reliance on physical registers despite availability of digital tools. As a result, facility staff maintained parallel systems, recording data in physical registers along with the digital entry. During supervisory visits, paper registers remained the primary focus of verification, with supervisors documenting their observations directly in the registers, mitigating the incentive to use the digital systems.

Underlying tensions were observed between care giving and reporting requirements. Health workers typically spent the first half of the day on conducting OPDs, and the second half on data related work and the conducting of lab tests. Pharmacists reported that not all clinically symptomatic cases identified during OPD were entered into the presumptive “P” form, explaining that *we have not got the instructions to report all the cases in the ‘P’ form. If they ask, then they have to give extra staff to do data entry for it.”* Another pharmacy officer noted that “*If any of the cases are found positive during the lab test, then we report that case in the ‘P’ form if it has not been reported earlier*.

Pressure from senior officials, coupled with fear of reprimands or formal memos, contributed to underreporting. A lab technician at a sentinel hospital reported experiencing pressure from a block officer (who has given additional charge of DMO) to underreport positive dengue cases in his jurisdiction. One DMO described said: “*when we started dengue testing heavily, we found more dengue cases with and without symptoms. Then our state officials started scolding us due to the high number of dengue cases. In such cases, we adopted the middle path while reporting data.*” Monitoring practices further reinforced this behavior by prioritising the achievement of reporting targets over data quality or completeness (Take earlier) Dengue reporting took place through two parallel channels—NVBDCP and IHIP-IDSP—where NVBDCP relied largely on physical records or WhatsApp communication, which was uncoordinated with the digital IDSP channel.

## 4 Discussion

### 4.1 Linking structures to practices

**Figure 1:**
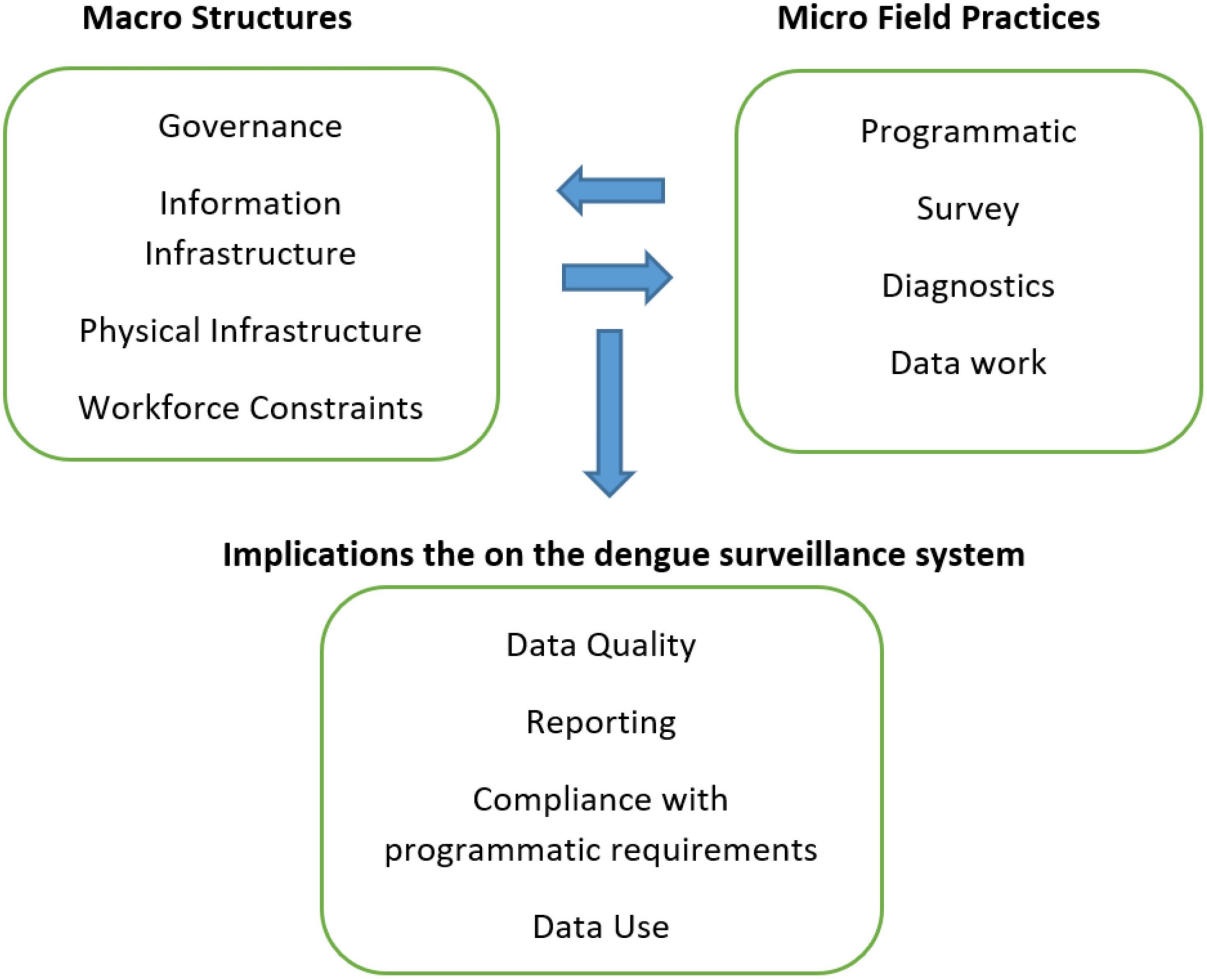
Macro–micro linkages shaping dengue surveillance outcomes.

The overall case analysis is presented as unfolding through the mutual interaction of the macro level institutional structures with the micro level surveillance practices, and their implications on the processes of dengue surveillance. While the macro structures represent conditions which are pre-existing to the context and relatively external to the dengue surveillance processes, micro practices represent what the staff do routinely and repeatedly in their everyday work in conducting surveillance. Our framework highlights that macro structures and practices are intimately inter-related, each shaping the other, and this entanglement influenced the trajectory of the dengue surveillance processes. Presented below is the schematic representation of this framework (see Figure 1).

#### 4.1.1 Structure-practices relationship

##### 4.1.1.1 Governance-programmatic practices

Governance defines the institutional framework, guidelines, overarching policies, and frameworks for defining budgets, responsibilities and reporting requirements (47). In India, governance of communicable diseases like dengue is a shared responsibility of the central and state governments under the Concurrent List (List III) of the Seventh Schedule of the Constitution. The governance architecture functions at three levels, with the centre primarily responsible for providing policy frameworks and financial support, and state and district levels tasked with translating these frameworks into operational routines within the health system. While this framework is mandated in theory, in practice the centre because of the hierarchy and funding powers, wield undue authority over defining the state level operational decisions.

Our analysis highlighted two independent national programmes for dengue—NVBDCP and IDSP, each with their own systems of governance. These systems often did not operate seamlessly due to their distinct operational logics in institutional arrangements, workforce, infrastructure and reflected in divergent norms such as for outbreak detection criteria. There was the dominance of one programme over other in practice, resulting in parallel and sometimes conflicting data workflows, resulting from respective programmatic practices, around reporting, testing, conduct of surveys, and accessing resources such as for transportation.

We provide some examples of these variations from our case study. NVBDCP followed static outbreak criteria while IHIP-IDSP criteria was based on data reported for three previous years. Entomological data—encompassing larval and adult mosquito surveillance by MPHW(M) and Insect Collectors—were submitted to NVBDCP via Excel spreadsheets and physical forms yet remain unintegrated with the epidemiological data captured in the IHIP-IDSP. Platelet counts—a key dengue diagnostic marker derived from complete blood count tests via state-contracted laboratories—were institutionally decoupled from both surveillance streams, perpetuating informational silos.

##### 4.1.1.2 Information infrastructure-data work practices

Information infrastructure refers to the heterogenous networks that link human and non-human entities (48), including hardware, software, networks, paper artefacts, staff, institutional practices, testing equipment and more. These networks support the collection of data, its quality assurance, and analysis. In dengue surveillance, this infrastructure also comprised of user manuals, standard operating procedures, embedded coded checks and balances, leading to hybrid digital and paper-based reporting systems.

Data work practices encompass the situated ways in which actors in their everyday work produce, quality assure, analyse and use data drawing upon and contributing to the stabilization or not of this information infrastructure. Some specific informational components of this infrastructure included the protocols for target-based monitoring, informal reporting channels, clustering criteria for symptomatic dengue case detection, locally variable practices of conducting and interpreting diagnostic tests, and routine prioritization of patient care over timely data entry and reporting. These practices help to understand how health workers’ perceptions prioritize data as a means to make their activities visible to higher-level officials rather than as a resource for supporting local, data-informed action. Institutional pressures, arising through reprimands for not meeting reporting targets or reporting dengue related deaths, encouraged health staff to resolve issues locally instead of escalating them through formal reporting.

The operations of two parallel information infrastructures governed by NVBDCP and IHIP-IDSP respectively, becomes a source for ambiguity in the shaping of data practices. The NVBDCP emphasized community level fever surveys and vector specific entomological surveys and manual reporting while IHIP-IDSP emphasized the digital case-based reporting of syndromic presumptive and laboratory data. This differing focus is reflected in the data practices. Inadequate digital infrastructure and workforce constraints coupled with target-based monitoring influenced how quality checks are coded in IHIP-IDSP software and the generation of automated analytics and reports, reinforcing target-based performance metrics and indicators.

This coexistence of digital and manual reporting channels shaped the perception of health staff regarding digital data as a means to creating visibility to higher officials rather than as a source of building local informed action. The NVBDCP, as an implementing agency, focuses on sentinel hospital confirmed tests, while marginalizing RDTs logged in IHIP-IDSP “L forms” despite their diagnostic value. The use of WhatsApp – an informal digital application over formal digital reporting from both private and public health facilities, undermined adherence to formal digital reporting pathways, yet simultaneously enabling faster communication and more rapid outbreak response. This raised the dilemma for policy makers for institutionalizing these tools which enable effective local communication but may undermine accountability made visible through formal digital surveillance networks.

##### 4.1.1.3 Physical infrastructure—survey and diagnostic practices

Physical infrastructure refers to the material resources, facilities, and logistical arrangements that enable programme functioning, such as in conducting surveys, doing diagnostic test and reporting. In dengue surveillance, this also included computer and internet connectivity that shape reporting processes, availability of paper records and registers, transportation facilities to conduct surveys, access to hospitals and labs and availability of diagnostic kits, water supply and maintenance support, and access to sentinel hospitals. These material resources condition what kinds of survey and diagnostic activities are practically feasible, where, and with what intensity.

The physical infrastructure is an important component of the information infrastructure and a crucial prerequisite to make the entire system work. For example, the availability and access to the physical infrastructure shapes how survey and diagnostics practices are carried out and how frontline staff operationalize surveillance and response activities under given material constraints. These include conducting surveys only in logistically convenient areas, negotiating community resistance, leveraging gram panchayat interventions, creating backup ELISA testing arrangements at neighbouring facilities, and often forcing the use of personal resources for data reporting. In our case study, we saw sentinel hospital laboratory technicians autonomously reprogramming ELISA equipment at proximate blood banks as contingency measures when primary hospital machines malfunctioned. This workaround helped mitigate diagnostic delays in dengue testing workflows and highlighted a critical reliance on informal networks to sustain service continuity amidst unreliable core infrastructure. Laboratory technicians resource pooled for timely maintenance servicing of ELISA equipment in conditions of inadequate maintenance support.

The status of the physical infrastructure and its gaps prompted staff to tactically adapt programme requirements to local realities, balancing formal expectations with time, resources, and access constraints. Deficits or interruptions in physical infrastructure directly shape and sometimes distort survey and diagnostic practices. For example, lack of transport or staffing of inspect inspectors led to the systematic skipping of remote villages in mosquito surveys, while the absence of an automated ELISA washer necessitated additional manual steps to counter the incorrect results arising from insufficient washing. The lack of standard operating protocols and supervisions also contributed to variations in the conducting and interpreting confirmatory diagnostic tests.

At the same time, compensatory practices—such as involving gram panchayat members to address community resistance, using personal mobile phones and internet for data reporting, purchasing registers for manual documentation, and working extra hours during outbreaks—demonstrated how frontline actors absorbed infrastructural gaps to keep the system functioning, albeit in ways that were uneven, unsustainable, and difficult to standardize or monitor. These local improvisations in responding to infrastructure deficits, keeps the show going.

##### 4.1.1.4 Workforce constraints—work practices

The Indian Public Health Standards specify that one PHC in rural areas should serve a population of 20,000 in hilly or tribal regions and 30,000 in plains, but in practice these norms are violated with workers practically catering to much larger numbers. This overstretch is compounded by persistent shortages of frontline workers, laboratory technicians, and doctors, which the field staff needing to find ways around, such as sharing staff across facilities and through multitasking. Undoubtedly, these structural workforce gaps shape the volume and quality of work shaping the dengue surveillance system.

In practice, workforce constraints translated into staff taking on additional responsibilities without supporting compensation that diluted attention to surveillance tasks, compromising data quality, follow-up, and responsiveness. At the same time, frontline workers actively developed local coordination strategies to mitigate these pressures. For example, the MPW (M) responsible for large catchment populations or holding multiple charges frequently coordinated with ASHA workers via WhatsApp or phone calls to obtain updates on symptomatic cases in villages where surveys could not be completed, thereby informally extending their reach. During outbreaks, staff support was temporarily pooled across neighbouring subcentres or PHCs to accelerate response activities, illustrating a form of *ad hoc*, and horizontal resource sharing based on mutual collaboration and solidarity.

Workforce shortages also prompted collective arrangements for laboratory-related tasks. Blood samples destined for distant sentinel hospitals were often aggregated across nearby facilities, with one MPW (M) from each facility jointly transporting specimens on a weekly basis, thus economizing limited staff and travel times. These local workarounds helped sustain routine surveillance and emergency response under constrained conditions, relying heavily on discretionary effort, informal coordination, and personal commitment. These practices raise questions of sustainability, equity, and the invisibility of such practices in formal programme design and resourcing.

In the Table 4, we summarize key aspects of the interactions between institutional structures and micro level practices around dengue surveillance, which has overall implications on the conduct of the dengue surveillance system which is next discussed.

**Table 4:** Summary of field challenges and coping strategies.

Data-related theme	How it was experienced by staff	Copping strategies reported
Community-level surveillance	Community resistance, lack of transport facilities, lack of resources, ad hoc duties	Seeking support from the gram panchayat to deal with community resistance Convenient way of conducting surveys (Preferred only nearby villages for surveys, delays in conducting surveys outside mosquito resting hours) Limited indoor container survey on a routine basis. Lack of adherence to guidelines
Differential reporting practices and laboratory variations	Lack of Standard Operating Protocols (SOP)/refresher training, heterogeneous lab methods, lack of enforcement and monitoring	Subjective way of conducting lab tests and interpreting the lab results Varied practices of data reporting based on the coordination between health staff as well as knowledge about the guidelines. Data analysis performed for target monitoring
Limited value of digital tools	Daily WhatsApp reporting target chasing, review meetings centered on coverage	Preference for data reporting over data quality and data completeness Lack of use of data for local action Informal mode of reporting Differences in physical register and digital reporting
Difference between physical realities and digital reporting	difficulty in detecting asymptomatic cases, resources constraints, pressure to under-report, lack of mandatory guidelines, lack of private reporting	Preferred to undertake fogging, spraying, and mass drug administration tasks over-reporting Prioritized patient care over data reporting Nil reporting Reporting only a few cases from the OPD

#### 4.1.2 Implications of structure-practice interactions on dengue surveillance system

##### 4.1.2.1 Data quality

The configuration of the surveillance system and how it is enacted in practice has substantial implications on data quality, understood in terms of accuracy, completeness, comparability, and timeliness. Fragmented governance, parallel programme-specific reporting systems, and the partial integration of third-party laboratory services contributes to inconsistent and incomplete datasets, particularly for febrile and dengue-suspect cases. The coexistence of digital and manual reporting channels, heterogeneity in RDT kit brands and their use practices, variation in ELISA procedures, and the systematic omission of remote villages in mosquito surveys, compound variability in process of data generation and recording. Target-driven monitoring reinforces a focus on achieving numerical targets and producing performance-oriented indicators, often at the expense of careful verification, representativeness, and timely updating of surveillance data.

##### 4.1.2.2 Reporting systems

The design and operation of dengue surveillance shape the nature and reliability of reporting systems. Multiple centrally designed programmes with distinct outbreak detection criteria, and reporting workflows produce parallel streams of information, increasing duplication, creating misalignment and expands work to harmonize diverse datasets. While the use of informal reporting channels such as WhatsApp and phone calls improves the speed of communication to support faster outbreak response, they simultaneously weaken adherence to formal reporting pathways, leading to uneven documentation and gaps in official records. These challenges are compounded by shortages of staff, transport, and basic infrastructure, which enhance selective or delayed reporting—for example, skipping hard-to-reach villages, under-reporting of OPD cases or batching sample transport and corresponding data entry. These workarounds prioritize clinical responsibilities over meeting routine reporting obligations.

##### 4.1.2.3 Compliance with programmatic requirements

Workforce and infrastructural constraints, combined with complex governance arrangements, influence the extent and nature of compliance with formal programme requirements. Overlapping structures and non-standardized policies, impede enforcement and supervision particularly regarding varying RDTs and diagnostic protocols, challenging frontline workers to adhere fully and consistently to all specified guidelines. Facilities serving populations far beyond Indian Public Health Standards and experiencing chronic manpower shortages rely on staff to hold multiple responsibilities, resulting in partial or negotiated compliance in which some surveillance tasks are adjusted, compressed, or informally delegated.

##### 4.1.2.4 Data use

Finally, the configuration of dengue surveillance has important implications for how data are used in practice for strengthening surveillance system, particularly for mitigating the dengue threat in local communities. For many frontline workers, data are perceived primarily as instruments for upward accountability and visibility, to demonstrate activity and performance to higher-level officials rather than serving as a resource for locally grounded, data-informed decision-making. Weak integration between routine surveillance systems, third-party laboratory data, and programme-specific data flows constrains the generation of comprehensive, timely analytics that could inform targeted interventions. At the same time, rapid operational decisions, particularly during outbreaks, often rely on informal communication networks and tacit local knowledge rather than the systematic analysis of surveillance data, resulting in formal databases being underutilized for strategic planning and local action.

### 4.2 Contributions

While a majority of interventions in the dengue surveillance system are largely biomedical in nature, our analysis highlights the gaps in existing biomedical interventions as well as highlight the equal importance of social, cultural and environmental factors. Existing syndromic surveillance is largely oriented toward malaria while little attention paid to other arbovirus diseases. The study conducted in one of the malaria-endemic regions in India showed that, among 260 samples that were negative for malaria by RDTs (192 collected from the community and 68 from a PHC), 23% tested positive for other infections, including 31% for dengue, 19% for chikungunya, and the remainder for other or mixed infections (49). A recent systematic review of the causes of acute undifferentiated febrile illnesses in the Asia region revealed that a significant proportion of fevers were attributed to dengue, typhoid, and other vector-borne diseases (50). This diversity highlights the need to scale attention to other non-malaria febrile illnesses to determine their prevalence and estimate the actual burden (49–52). It is also essential to strengthen the knowledge of the frontline workers about other vector borne diseases, as well as include more symptoms in the current syndromic surveillance for differentiating febrile illnesses.

The challenges in community level surveillance such as community resistance was also found in developed and developing countries including Singapore, Australia, the United States, and Taiwan (53). Despite these constraints, larval surveys remain a cornerstone of community-based surveillance, even though their capacity to reflect actual transmission dynamics is limited (54). To overcomes this, countries have combined enforcement along with participatory education to sustain vector control (53). In addition to larval surveys, countries have also used newer technologies for capturing larval indices, such as autocidal ovitraps, gravid female traps, hand-held terminals (HHTs), and geographical information system (GIS) technology. Since the role of adult female mosquitoes is crucial in disease transmission, countries have used various methods for tracking the adult vector population (54). which is particularly useful in countries experiencing shortage of expertise and manpower in dengue vector management.

Epidemiological surveillance faces similar challenges. Varied reporting practices, lack of laboratory standardization, resource constraints, and minimal private sector involvement collectively weaken data quality and timeliness. The Asia Pacific and Americas Dengue Prevention Board previously emphasized the need for standardized confirmatory testing and highlighted the limitations of centralized laboratories, which delayed results for clinicians (25). Persistent barriers, including the cost and technical demands of diagnostic tests, reinforce the importance of affordable point-of-care diagnostic kits and greater integration of private-sector data to reduce underreporting (25, 55). Underreporting also has political dimensions to appease public concerns and mitigate pressure on the administration by artificially minimizing the perceived scale of the disease outbreak (56). Addressing these issues requires systemic reform to strengthen India’s understaffed primary healthcare system and improve data reliability at the periphery, which is crucial for evidence-based disease control efforts (57).

Our findings also highlight that dengue control and prevention efforts were largely static, primarily focusing on chemical-based interventions, source reduction, and community-based awareness campaigns, despite their limited effectiveness. The absence of formal mechanisms for intersectoral collaboration hinders the development of a comprehensive, long-term prevention strategy. Similar studies conducted in endemic countries have also demonstrated a consistent lack of systematic, data-driven approaches for identifying and responding to the early stages of outbreaks (55). The need for an integrated surveillance system that combines data from diverse sources and dimensions is paramount for risk stratification, as well as to provide early warning signals (62). However, these disparate data sources remain dispersed across different organizations, rendering their aggregation and comprehensive data analysis difficult. We observed a significant lack of integration between entomological and epidemiological components, with separate reporting channels maintained for each through different implementing agencies.

To foster the integration, the involvement of experts from various departments is essential to gain diverse perspectives (16). Additionally, the availability of data from different sources such as satellite and remote sensing data for understanding vegetation density, rainfall, and surface temperature, national census to provide demographic information, such as population density, age distribution, housing structure, and the availability of piped water supply along with routine surveillance data helps to risk stratification as well as to provide early warning signals. Leveraging this data will help unify the social and biomedical conditions data into the surveillance system. Countries such Colombia, Mexico and China has taken efforts develop integrated system to provide early warning as well as planning, monitoring vector control interventions (17, 31, 58).

It is worth noting that most of these integrated data efforts are vector-centric, i.e., they utilize insights from the integrated system for vector control. Muurlink and Taylor (59) draw attention to the impact of the climatic factors and socio-economic factors on the host, i.e., humans, who also become vulnerable to infections through different pathways, for, e.g., the unfavourable climatic conditions impact agricultural productivity, which results in undernutrition among locals, which may be considered as a surrogate marker of impaired immunity to dengue infection in endemic areas. Socioeconomic factors, such as reduced access to nutrition, education, and protective measures, as well as a reduced ability to adjust work hours to avoid exposure, also lead to greater vulnerability to disease (59). Hence, it is essential to consider risk factors that impact both humans and vectors in the integrated surveillance system. Taiwan’s experience during COVID-19 illustrates how attention to micro-level practices, comprehensive databases, multi-sectoral collaboration, and flexible local adaptation can support effective pandemic prevention (60).

The WHO Global Vector Control Response (GVCR) 2017–2030 provides a guiding framework emphasizing intersectoral collaboration, community engagement, integrated vector control interventions, and robust monitoring and evaluations (61). Singapore’s success in achieving lower dengue seroprevalence demonstrates the potential of such an integrated approach, while the difficulties faced by resource-limited countries highlight the need for global funding and research investment to develop scalable, cost-effective solutions (6, 61).

## 5 Conclusion

The paper has presented an empirically based analysis of the interplay between institutional structures and microlevel practices and their implications on the dengue surveillance system. Macro level structures of governance, revealing how governance, infrastructure—information and physical, and workforce dynamics, systematically shape micro practices. This analysis highlights the complexity of the dengue surveillance system, which defies simple fixes. Often the frontline health workers are put to blame for the ills of the system, and remedial measures of more training or digital systems are proposed. However, our analysis highlights that bringing in improvements at the practice level are not easy to introduce as they are intimately interconnected with macro level institutional structures. Reforms in these structures and practices need to be taken in conjunction for making meaningful change.

The analysis also offers some technical recommendation to strengthen surveillance system. Current surveillance forms primarily capture clinical parameters, overlooking key transmission drivers. One concrete improvement would be to include mandatory environmental and sociodemographic fields within the S-form, such as water supply status, presence of waterlogging, construction or plastic waste, and recent travel history or mobility patterns. Incorporating these variables would enable more systematic analysis of transmission risk and support more targeted and context-specific response strategies.

Traditional policy frameworks highlight that innovations originate in the centre, and these need to be diffused to the field levels. This model undermines the potential for innovations being developed in the periphery. Our analysis highlights the tremendous potential which is situated in the field, and the pressures they experience in carrying out their everyday practices, such as of reporting and conducting diagnostic tests, force them to develop striking innovations. This was evident in the use of WhatsApp communication channels to speed up sharing of information, or pooling of resources and multitasking to deal with existing structural constraints. Rather than subjecting health workers to constant reprimand and blame, it is important to identify the potential for innovation, and how this can be fostered and scaled. This requires a shift in our policy making processes of “putting the bottom first”, implying taking seriously the concerns and work of the field staff, which will also lead to the improved design of digital systems that are better aligned to local work practices and contextual conditions.

The socio-technical infrastructure perspective adopted in this analysis highlights the interconnected and heterogenous nature of the overall system comprising dengue surveillance. This perspective then highlights the futility of making piecemeal interventions without taking the overall system and their interconnections into account. As is often the case, digital solutions are seen to be the panacea to address larger challenges which by definition are socio-technical in nature. Digital interventions without making changes in structures of governance or addressing workforce constraints are not likely to yield improvements to the overall surveillance system. Similarly, mandating testing norms without ensuring adequate access to diagnostics equipment or transportation facilities to conduct required surveys, are going to be ineffective.

Recognizing dengue as an environmental and social disease requires a shift from biomedical control toward a socio-epidemiological framework that integrates environmental, behavioral, and digital data. Our results highlight how social determinants, such as water scarcity, inadequate waste management, and poor housing, amplify vector breeding and outbreak risks. Strengthening surveillance must include social factors in equal measure and develop socio-technical approaches to foster interoperability and data sharing across biomedical and social data to develop more unified perspectives to strengthen surveillance. The bio and social conditions shaping dengue represents two sides or the same coin and must be taken in unison.

This study has limitations. First, the study was conducted in selected districts within a single Indian state, which may limit the generalizability of the findings to other epidemiological and health system contexts across India. However, the study provides higher level analytical insights into studying information and work practice related flows that may be transferable to similar settings, with appropriate contextual adaptation.

Second, while the private sector plays a significant role in healthcare delivery in India, and its underreporting is recognized as a major challenge, this study focused exclusively on public health facilities. This was a deliberate choice to enable an in-depth examination of the public health system as the primary backbone of surveillance and outbreak response. We acknowledge such a focus to be limiting and ignoring the challenges of integrating data from the private sector to that of the public system. We highlight this as an important area for further research.

Finally, the selection of a “high burden” district for the case study may have introduced a degree of selection bias, potentially influencing the observed practices. In addition, as with all qualitative research reliant on open-ended responses, there is an inherent risk of limited scale, which was chosen over greater analytical depth. The lead author’s formal engagement with the State Health Department, while relevant for access and uptake of research findings, may have introduced a sense of bias in the research.

## Data Availability

The original contributions presented in the study are included in the article/supplementary material, further inquiries can be directed to the corresponding author.

## Footnotes

1 House index: percentage of houses infested with larvae and/or pupae. HI provides information on extent of breeding. It is calculated as HI = (Number of houses infested/Number of houses inspected) × 100.

2 Container Index (CI): percentage of water-holding containers infested with larvae or pupae. CI provides information on intensity of breeding. It is calculated as CI = (Number of positive containers/Number of containers inspected) × 100.

3 Breteau Index (BI): number of positive containers per 100 houses inspected. BI is a yardstick for evaluation of the control strategy. It is calculated as BI = (Number of positive containers/Number of houses inspected) × 100.
